# Developments in Australian general practice 2000–2002: what did these contribute to a well functioning and comprehensive Primary Health Care System?

**DOI:** 10.1186/1743-8462-3-1

**Published:** 2006-01-15

**Authors:** Gawaine Powell Davies, Wendy Hu, Julie McDonald, John Furler, Elizabeth Harris, Mark Harris

**Affiliations:** 1 Centre for General Practice Integration Studies, School of Public Health and Community Medicine, University of New South Wales, NSW 2052, Australia; 2 Julie McDonald & Associates, PO Box 98, Jamberoo NSW 2533, Australia; 3 Department of General Practice, University of Melbourne, Victoria 3010, Australia; 4 Centre for Health Equity, Training, Research and Evaluation, School of Public Health and Community Medicine, University of New South Wales, NSW 2052, Australia

## Abstract

**Background:**

In recent years, national and state/territory governments have undertaken an increasing number of initiatives to strengthen general practice and improve its links with the rest of the primary health care sector. This paper reviews how far these initiatives were contributing to a well functioning and comprehensive primary health care system during the period 2000–2002, using a normative model of primary health care and data from a descriptive study to evaluate progress.

**Results:**

There was a significant number of programs, at both state/territory and national level. Most focused on individual care, particularly for chronic disease, rather than population health approaches. There was little evidence of integration across programs: each tended to be based in and focus on a single jurisdiction, and build capacity chiefly within the services funded through that jurisdiction. As a result, the overall effect was patchy, with similar difficulties being noted across all jurisdictions and little gain in overall system capacity for effective primary health care.

**Conclusion:**

Efforts to develop more effective primary health care need a more balanced approach to reform, with a better balance across the different elements of primary health care and greater integration across programs and jurisdictions. One way ahead is to form a single funding agency, as in the UK and New Zealand, and so remove the need to work across jurisdictions and manage their competing interests. A second, perhaps less politically challenging starting point, is to create an agreed framework for primary health care within which a collective vision for primary health care can be developed, based on population health needs, and the responsibilities of different sectors services can be negotiated. Either of these approaches would be assisted by a more systematic and comprehensive program of research and evaluation for primary health care.

## Background

In Australia, as in other developed countries, demands for healthcare services are increasing. This is fuelled by longer life expectancies, technological advances and high consumer expectations, and constrained by limited budgets and increasing health care costs. The burden of disease consists increasingly of chronic conditions, which are unequally shared across socio-economic groups [1].

Primary health care (PHC) has been advanced as a cost-effective way to improve health outcomes in an equitable manner [2], with some supporting evidence from cross-country comparisons [3,4]. Much recent activity has sought to strengthen the role of general practice in the Australian primary health care sector and its links with other services [5]. However there is still some disagreement about the role of general practice within Primary Health Care, linked in part to ongoing debates about the scope of primary health care [6]. This has contributed to a dissonance between the broad thrust of program and policy initiatives and the underlying conceptual debates, and created an uncertain environment for the development of a comprehensive PHC sector and of a supporting research and development agenda.

The aim of this study was to review the extent to which national and state/territory level initiatives (as at 2002) that were directed at strengthening the contribution of general practice to primary health care were contributing to a more comprehensive and well functioning primary health care system. This was assessed against a conceptual model of the essential aspects of a primary health care system, based on previous work [5], using information from program and policy documents, published evaluations and interviews with staff responsible for these programs together with the perceptions of those involved in coordinating the implementation of the programs at national and state level. The aim of the study was to give an overview of developments, rather than evaluate specific initiatives. Details not reported here can be found in the full report at 

There are differing views on the definition of the terms primary health care and general practice [7]. For the purposes of this paper, primary health care is taken to include general practice and state funded generalist community health services, private allied health services and pharmacies and complementary therapists (although not all of these were involved in government initiatives during the study period), but not specialist or acute care outreach services. General practice is taken to include general practitioners and their practice staff, the practices from which they work and the Divisions of General Practice.

We used a normative model of PHC in our consultations to map the focus of initiatives and identify areas that have been relatively under-developed. For further details of the model see Additional File 1: Conceptual framework for a primary health care system. The model identifies Primary Health Care as having the following key elements:

• the broad *goals and values *that underpin the work of primary health care organisations, and the services and initiatives to which they contribute;

• the *service planning and development functions *required to develop and maintain effective services, including setting priorities, clarifying roles and responsibilities within and between services, and developing service plans;

• the *core functions *or types of service which primary health care services provide to meet the health needs of the population, including services to individuals and to populations;

• particular *approaches *or ways of providing or organising health care which are characteristic of primary health care and are often believed to contribute to its effectiveness;

• the *capacity *required for effective, adaptable and sustainable services;

• key *outcomes *and *indicators *that are relevant to primary health care systems

• *contextual issues *that effect how primary health care operates and its opportunities for development.

Each of these elements is characterised by a number of components, elaborated in additional file 1.

## Methods

Data were gathered and analysed between November 2002 to February 2003. Methods included a literature search and document analysis, semi-structured telephone interviews, workshops and an expert panel review. Findings were confirmed through triangulation, feedback to participants in repeat interviews and workshop presentations. The work had five phases.

### Modelling a comprehensive and well-functioning primary health care system

A normative model was constructed from a literature review and from repeated consultations with an expert panel assembled for this project. Members of this panel comprised general practice and primary health care academics and an external consultant. The model also drew on a recent wide ranging consultation on the core functions of primary health care, conducted by a panel member with NSW Area Health Service staff [8]. The model was circulated for comment to all those participating in the research, and then used to structure the interviews and program analysis.

### Pilot interviews of opinion leaders

The critical issues facing the primary health care sector as a whole were identified through 19 semi-structured interviews with individuals across Australia from Commonwealth and State/Territory health departments, State Based Organisations of Divisions of General Practice (SBOs) and academia. These persons were selected for their contribution to primary health care development in Australia through their research, publications or current work.

### Program and document scan

Policy and program documents were identified from interviews and searches of national and state/territory health department websites, and from the list of programs reviewed for a previous project [5]. Documents were included if the stated intention of the program to which they related was to strengthen the capacity of general practice to carry out its primary health care functions (including linking with other primary health care services). Initiatives that might contribute indirectly to this (such as workforce programs or changes to medical education) were not included. Programs were sorted according to the main stated focus of the initiative, and the documents scanned to assess the potential impact of major national and state/territory initiatives.

### Semi-structured interviews of key informants

Perceptions of the impact of recent initiatives on the primary health care system were assessed through semi-structured telephone interviews based on the normative model. These were conducted with 84 participants, either individually or in small groups convened by the initial contact person. Interview participants were selected for their potential to provide rich data from direct involvement with initiatives at a policy and program level, a standard qualitative sampling strategy [10]. They included managers of major national programs from the Commonwealth Department of Health and Ageing (DoHA), representatives from the state offices of DoHA, SBOs, and senior State/Territory health department staff responsible for primary health care from each State and Territory. Additional interviewees were identified by snowball sampling. Interviews were not conducted with GPs and other direct patient care providers as the focus of the study was at the level of policy and programs.

### Workshops and teleconferences

Two workshops (in NSW and Victoria), and two teleconferences (NSW and Queensland) were conducted to review and consolidate the findings and to gather new information, particularly regarding the nature of the relationships between different primary health care providers and organisations. The workshops were attended by 15–20 participants from a range of state based primary health care provider organisations and health department representatives.

### Analysis

Elements of the conceptual model were used as theme categories to analyse the extent to which the identified programs were contributing to the different elements of the primary health care system (see above).

Interview notes were thematically interpreted using Nvivo software for coding, sorting and retrieving data. Analysis involved the identification of common themes from within the data theme categories and the scan of program documents. The analysis was led by one of the authors (GPD) but also included the other contributing authors, who met to compare and reach consensus on common themes in the data. The focus groups and workshops were used to test the face validity of data analysis as it proceeded, and to refine the analysis.

## Results

Our findings are divided into two sections: the critical challenges facing the sector identified primarily from the pilot interviews, and the focus and impact of recent initiatives on the different elements in the model of primary health care, identified primarily from the other data sources.

### Critical issues impacting on the contribution of general practice to primary health care

We asked key informants about the critical contextual issues that influence the development of general practice and its relationship to the rest of primary health care. Three main issues were reported.

#### Changing patterns of health and health care demand

Increasing demand from an ageing population, a rising prevalence of complex and chronic conditions, high consumer expectations and technological advances were all identified as having a direct influence on developments in general practice and primary health care. These also put pressure on the acute care sector, which in turn puts pressure on general practice and primary health care. This pressure was seen as sometimes diverting primary health care from its core functions.

#### Systemic fragmentation

This was a consistent theme. Commonwealth and state initiatives were not seen as fitting well with each other, and there was a lack of integration between public and private sectors, acute and primary health care providers and within primary health care itself.

#### Insufficient capacity

This was seen to limit what could be achieved through general practice and other primary health care services, either separately or working together. Workforce was considered a particularly pressing problem, in allied health and nursing as well as in general practice itself. This limited the capacity for substitution of roles, although of course this does not mean that new categories of health professionals (such as the 'physician assistants' used in the USA) could not be developed. Systems for information management needed further development, and there was a lack of overall leadership, including the lack of a coherent national approach to primary health care.

These contextual issues provided the background against which other, more specific developments needed to be understood.

### The extent to which initiatives are contributing to a well functioning and comprehensive primary care sector

We identified a wider range of programs targeting general practice than in an earlier review [5]. Tables 1 and 2 show the range of national and state/territory programs reviewed, grouped by main focus.

**Table 1:** Main focus of national initiatives

Focus (Number of Programs)	Programs
Addressing general capacity issues (8)	Carelink (central contact point for information about a range of services) Divisions of General Practice Domiciliary Medication Management Review Enhanced Primary Care (payment incentives to GPs) More Allied Health Services (in rural areas) Nursing in General Practice PHC Research, Evaluation & Development Practice Incentive Program (payment incentives)
Addressing a specific condition (7)	Chronic Disease Initiatives: the National Integrated Diabetes Program and Asthma GP Immunisation Incentives Mental health strategies, including Better Outcomes in Mental Health and Primary Mental Health Care National Drug and Alcohol strategies including the Illicit Drug Strategy Rural Chronic Disease Initiatives Screening: breast and cervical cancer Smoking, Nutrition, Alcohol & Physical Activity
How services are organised for populations (5)	Aged care After Hours Primary Medical Care Coordinated Care Trials PHC Access Program (remote areas) Regional Health Strategy program
Treatment modalities (2)	National Prescribing Service initiatives Sharing health care (self management)

**Table 2:** Main focus of State/Territory initiatives

Focus	Initiatives (Number of jurisdictions)
Building capacity and planning	Memoranda Of Understanding between DGP & State/Regional health departments (6) PHC related policies (5) State-wide plans, reviews (5) State level GP liaison/adviser (2)
Addressing a specific condition	Mental heath (5) Chronic disease initiatives (2) Prevention initiatives (1)
Tools/communication	Registers, directories (2) Call centres (2) Information management (2)
Working together locally	Primary Care Partnerships (1) and Networks (1) Co-location of services (1)

National programs tended to establish discrete initiatives, many of which related to specific health issues (eg asthma) or to building capacity within general practice (eg the Divisions program). Most states and territories had or were developing primary health care policies, as well as being involved in policies, plans and agreements to create links with national programs and coordinate implementation at state or regional level. While these appear to be complementary approaches, informants reported that their experience was of parallel streams of service provision with some scattered links between them rather than any integration of national with state level initiatives. This left regional and state level organisations with the role of integrating disparate programs to meet needs at those levels.

Table 3 takes four of the elements of the model of primary health care and summarises those which appeared to have been best and least well served by recent developments.

**Table 3:** Aspects of the primary health care system best and least well served by recent initiatives

PHC Model aspect	Components best supported	Components supported to some extent	Components least supported
Functions	Ongoing care for chronic and complex conditions Facilitating and coordinating care	Prevention, early detection and risk factor management Episodic care (eg After Hours care) Strengthening community capacity (largely through indigenous programs and the Regional Health Strategy)	Healthy public policy (although this is addressed to some extent through the Divisions' movement) Safe and healthy environments Promoting normal development
Approaches	Multidisciplinary care	Multiple and systematic strategies Evidence based Building consumer capacity Working across sectors	Working within a socio-ecological framework
Capacity	GP workforce Leadership Planning and collaboration processes	Local and State organisational structures and partnerships IT/IM systems Service planning GP patient access to allied health and consulting pharmacy services	Coherent P HC Policy across Commonwealth and State Training in team work, especially across organisations Monitoring, QI & evaluation
Indicators	Workforce distribution Utilisation of PHC services	Accredited practices GP chronic disease indicators	Access Sustainability

The *function *which had been best supported was chronic disease care, especially the role of general practice in care coordination. This was in the process of being extended to prevention and risk factor management through the work of the then Joint Advisory Group on Population Health [11]. There had been some attention to developing community capacity (particularly in rural areas) and to improving access to episodic care, particularly after hours and for underserved rural areas, but there was little emphasis on developing healthy public policy or safe and healthy environments. The *approaches *reflected these functions: considerable emphasis on multi-disciplinary care for individuals, but rather less focus on more comprehensive approaches such as multiple and systematic strategies, working across sectors or working within a socio-ecological framework. Informants identified the lack of strong links between consumers and providers as a particular problem here. Some identified promoting appropriate consumer demand through education and incentives (for example immunisation incentives for parents and carers) as a promising but little used approach.

*Capacity building *was focused chiefly on general practice, with new forms of payment to support extended roles, considerable attention to workforce and leadership within the sector and processes for planning and collaboration around national initiatives. There had also been some improvement in access to allied health and consulting pharmacy services for GP patients. There had been less attention to structures and partnerships for integrating Commonwealth and state funded initiatives at state and regional level, and least focus on core elements of an integrated system of care: a policy framework, indicators, systematic quality assurance and evaluation and development of a strong evidence base. There had also been little focus on training in team work, or developing the capacity of other primary health care organisations such as community health services, consumer and community groups.

A number of barriers to better *service planning *were identified, and many informants in both the pilot and key informant interviews noted that fee-for-service payments did not properly support population health activities and chronic disease care. There were problems in focussing too strongly on general practice, since some tasks might at times be better performed by other professionals. Some Commonwealth programs were 'silo-ed' and had short time frames, making it difficult to coordinate activities or pool resources at state and regional level, or transfer the capacity gained in one program to its successor. Many saw these problems as reflecting the way that responsibility for health care was split between different levels of government. The resulting fragmentation left many interviewees understanding little of primary health care beyond the areas where they were directly involved. There had also been little progress in engaging consumers in planning, despite the expressed intentions of many interviewees to do so, and a number of clinicians expressed the view that planning did not seem to address the real problems that they experienced in their work.

Training continued to occur largely within professional and/or organisational boundaries, thus missing opportunities for developing multidisciplinary teams. Thus the education to support implementation of the Enhanced Primary Care program did not address multi-disciplinary care for general practitioners and engaged other primary health care practitioners only to a very limited extent [12]. However the Building on Quality program [13] did enable a number of Divisions of General Practice to develop innovative methods of small group learning, and the Primary Health Care Research, Evaluation and Development program [14] had a capacity building component with the potential to involve a wider range of professional groups.

Although information systems for general practice had developed considerably, this was not matched for many State community health services (with the exception of the jurisdictions involved in the development of the Community Based Health Information System) and there had been little attempt to link the two, except in the chronic disease registers established in some areas and the information systems used to facilitate care planning in the Coordinated Care Trials [15]. This important element of infrastructure for integrated service planning remained underdeveloped.

There had been some progress towards developing system wide quality indicators in general practice. However this remained narrowly focused around a few diseases (diabetes, asthma, mental illness) and a limited number of interventions (immunisation, prescribing, care planning).

Informants noted the lack of evidence to guide the choice of primary health care strategies. Although most major programs had involved literature reviews, evaluations had often been set up late, and there had been little formative research. However there were some examples of the good use of evaluation such as the After Hours Primary Medical Trials which evaluated strategic pilot programs and used this information to guide wider implementation [16]

Overall, many informants saw themselves as trying to weave a series of separate initiatives into a sustainable approach to primary health care. Having short term programs, often with a very specific focus, made this particularly difficult, as did the problems of working across the Commonwealth/state divide. There were some starting points for more systematic approaches, including the Bilateral Agreements (in population health and community care) between Commonwealth and State governments, and some regional and service delivery organisational networks. However collaboration was voluntary at each level, leaving the effectiveness of these frameworks hostage to factors such as the relationship between the Commonwealth and state health departments and State Based Organisations, the ability of Divisions of General Practice to integrate different programs at regional level, and informal relationships between service providers.

A strong theme that emerged in many of the interviews was that the time had passed for fragmented programs which focussed on single issues or worked only within one health care sector. Many respondents indicated that unless the systemic problems of fragmentation, lack of capacity and absence of a coherent approach to primary health care were addressed, then more narrowly targeted programs could have little sustained effect.

## Discussion

The project team was able to collect the views of a large number of people who are currently engaged in primary health care reform (103 by interview and 35 by workshop). Selection bias was minimised by systematically sampling senior persons from each jurisdiction and each type of organisation, and our sampling strategy aimed at identifying information rich informants. We specifically excluded consumers and service providers who did not also have program responsibility, given the focus of our study. We also did not focus on Aboriginal and Torres Strait Islander organisations. Recent reviews of primary health care in Aboriginal and Torres Strait Islander communities have been reported elsewhere [17].

The study was restricted to major national and state/territory wide initiatives. We did not review local and regional programs. Although these can be the source of important innovations, they have limited ability to trigger systemic change or mitigate the effects of fragmentation higher in the system.

Our research was centred on the extent to which programs aimed at strengthening general practice were contributing to better functioning and more comprehensive primary health care. Because there had been very few evaluations, judgements about the potential impact of programs were based on an analysis of their aims, methods and budgets, together with the perceptions of those interviewed as to their impact in the field. The normative model of primary health care, drawn from international literature, provided a framework for the study, allowing us to explore some of the tensions involved in and between these initiatives, and some of the barriers and facilitators to their success. There are, however, a number of other issues that impact on primary health care that are not reflected in the model, including competition between different parts of primary health care (for example, for funding or scarce staff), the changing demographics of the primary care work force, and shifts in the boundaries of professional roles.

The initiatives we examined were focused on general practice. This reflects the focus of current national approaches to primary health care development and is, as some respondents indicated, itself symptomatic of a divided system where there is no authority responsible for primary health care as a whole. The study also reviewed general practice and primary health care independently of their relationship to the rest of the health care system, including links with secondary and tertiary care. These external relationships can both promote and constrain the development of primary health care: for example, pressure from acute care can distract from the broader health needs of the population.

### Balanced reform?

Compared to our previous review [5], there was a greater emphasis on large-scale programs and some focus on system change. This reflects a growing acknowledgment at Commonwealth and State/Territory levels of the potential contribution of primary health care. However the investment was often short term and appeared unbalanced when compared to the model of Primary Health Care. A relatively narrow range of functions (areas of service provision) were being targeted, particularly chronic disease management. The focus was on developing general practice, with much less emphasis on links to or complementary developments in other parts of primary health care. This has the potential to undermine integration across primary health care and reduce the reach and effectiveness of some of the general practice initiatives, where these depended upon other parts of primary health care (eg state funded or private allied health care). Finally, there was little investment in overall primary health care system capacity or emphasis on system reform. As a result, while most respondents welcomed the increased investment in chronic disease care, they found that much of their effort went into overcoming the effects of systemic problems. More balanced reform would allow initiatives targeted at general practice to have a broader impact on the primary health care system. They would work across primary health care, addressing systemic problems and developing system capacity as well as building up specific areas of primary health care practice.

The research highlighted a number of areas in which primary health care lacks the capacity to develop as a coherent sector, which in turn limits the impact of the GP targeted programs we studied. These include leadership (which is currently fragmented across professional groups and government sectors), information systems to underpin more coherent operations and support coordination of care, and workforce development. Even with the addition of Service Incentive Payments, the fee for service system provides limited incentives in crucial areas such as population health. Practice Incentive Payments provide some support for capacity building or collaboration, within primary health care or with secondary care, but these have tended to be narrowly focused and to represent 'success' payment for achieving capacity rather than funds to invest in building that capacity. More recent developments (such as Medicare Plus) have begun to make MBS payments available for providers other than doctors, but these remain within the fee for service framework. It is worth noting that in the UK, New Zealand and Canada, primary health care reform has been accompanied by significant new funding across the sector. In Australia there have been substantial investments but these have continued to be focused on general practice rather than more broadly developing primary health care more broadly. This has in part been due to the Commonwealth/State division of responsibility in health care.

### Collective vision and a national framework

A strong primary health care system will require a coherent approach to investment, capacity building and service development, based on a common understanding of the scope and purpose of the sector. Our findings suggest that this is not reflected to any great extent in the programs for developing general practice included in this study. There are a number of possible reasons for this. A fragmented health system tends to limit interaction between different parts of primary health care (including different professional groups), and so limit the growth of mutual understanding. There is no single authority or common accountability for the health of defined populations, as in the UK and New Zealand. Policy makers and practitioners often appear to have very different views, and much of primary health care rests with largely autonomous service providers, with limited commitment to population health, needs based resource management, shared service development and clinical governance across professional groups. Health services reform has to be accommodated within short political and budgetary cycles which may vary across national and state governments. A shared vision for primary health care may make it easier to achieve systematic reform within these limitations. Such a vision will require a collective approach that includes all the major stakeholders, including consumers.

This will not be easy to achieve. There are differing views about the scope of the primary health care sector. Recent stakeholder consultations have revealed three views of primary health care [8]. The first is a comprehensive primary care model centred around first point of contact care, including general practitioners, allied health professionals, pharmacists and community nurses. A second view, of community based health care across the continuum of care also includes specialist community services such as aged care, mental health, drug and alcohol, chronic disease and hospital in the home. This model reflects the demands of the acute care sector. The third is a broader intersectoral model that addresses the multiple determinants of health. In our study, general practice more commonly adopted the first view, with a limited number of individuals in each group expressing the third, more public health oriented view. The second view was not frequently voiced, perhaps because the study did not sample representatives from the acute care sector.

In our study, different views of primary health care were often reflected in differences in goals and values. These were usually not raised immediately, but emerged as differences between groups of primary health care practitioners were explored. Where they are not explicitly acknowledged, such differences can lead to mutual misunderstanding and limit the scope for collaboration. They can be difficult to resolve as they tend to reflect a person's position and experience in the health care system, as well as their and professional and cultural origins. Some preliminary work using explicit stakeholder negotiation is occurring in Western Australia and Tasmania, but there is still no process for developing a national consensus on the role of primary health care that takes into account community expectations, the views of general practice and other provider groups.

How, then, is a more coherent approach to primary health care to be achieved? One approach would be to use a model such as the one used in this research to develop a national framework, delineating the broad functions and approaches of primary health care, describing contextual factors such as the relationships of primary health care to the rest of the system (in particular public health, acute care and specialist clinical streams in the community), and defining a relevant set of outcomes and performance indicators for the sector as a whole. Such a framework could then provide the context for planning more balanced investment, better coordinating State level planning and local service development, and taking a longer term and more comprehensive approach to capacity building. Such an approach could ideally support the development of a national policy or strategy, but would also assist the more incremental approaches that are characteristic of Australian health services reform.

Within such a framework, organisational arrangements and relationships could be negotiated at each level and across levels, setting out accountability for agreed outcomes through clear governance arrangements. These outcomes would be defined in terms of the needs of the populations being served, with these needs becoming the common reference point for service planning and review at each level. One approach here would be to make the regional level the main focus, and to design national and state programs so as to support comprehensive and flexible planning at this level.

The framework would also provide a guide to capacity building within the sector, especially with regards to funding, workforce, training and information systems. Current funding arrangements do not provide the incentives or flexibility to support system reform. Many participants noted the difficulty of using fee-for-service payments to reward population health activities and chronic disease care. Service Incentive Payments linked to specific conditions were seen as 'tinkering around the edges' rather than building practice capacity. More flexible funding arrangements at service delivery and program level would permit more effective patterns of service provision and broader capacity development. Better developed information systems with linkage across organisations and sectors would also support greater integration.

However, developing a national framework would require greater leadership than is currently evident. At present political leadership is hampered by conflicting interests between Commonwealth and state jurisdictions. Commonwealth and states have very different agendas in relation to primary health care, with the states having an immediate interest in reducing demand on public hospitals, whereas the Commonwealth has a stronger interest in reducing costs through the MBS and the PBS. While there are leadership groups in some parts of primary health care – particularly in general practice – there is no strong leadership to represent the sector as a whole.

Consumer and carer organisations will need to have a significant voice in such developments, but they have limited resources and their engagement with the sector is often ad hoc. There is no single professional association that is seen as the lead organisation for primary health care. However there have been continuing efforts to engage consumer and community input into Divisions of General Practice, including these in the new Divisions performance framework. This will need to be replicated at other levels.

Although it was not the aim of this study to examine workforce shortages in any depth, it was repeatedly quoted by participants as a key critical issue. Multidisciplinary approaches were often suggested to improve the quality of care, and sometimes role substitution as a strategy to address workforce shortages. However the shortages in most health care professions may critically limit both strategies.

Finally, although there is broad consensus about many of the issues raised in the consultations, the evidence base is very limited. If primary health care is to be developed in a more systematic way, this will need to be supported by a coherent and well supported research and development agenda. The model used in this study and the comments of participants, provide one starting point for such an agenda.

## Conclusion

This study has highlighted limitations in recent developments in general practice that reflect the lack of a coherent approach to primary health care in Australia. It suggests that while recent initiatives have strengthened specific areas of general practice, they have been undermined by insufficient focus on strengthening complementary aspects of other parts of the primary health care sector, improving links across primary health care and developing the capacity needed to underpin sustained development. This has reduced the contribution of these initiatives to a comprehensive and sustained primary health care system. While this narrow approach is understandable in terms of the different levels of government and professional groups involved in different parts of primary health care, it creates significant difficulties for those trying to develop coherent and comprehensive primary health care services at state and regional level. The development of an agreed framework for primary health care would be a valuable step towards articulating a common vision for primary health care and would provide point of reference for planning more balanced developments, leading perhaps to a national policy and strategy for primary health care. It would also provide the basis for an agenda for research which would further develop the evidence base for primary health care.

## Competing interests

This study was funded by the Commonwealth Department of Health and Ageing. All the authors have undertaken other studies funded by the Department over recent years.

## Authors' contributions

GPD, WH, JMcD and JF conducted the study. EH and MH acted as senior consultants and provided particular input into the model used in the study and the interpretation of the results. All authors read and approved the final manuscript.

## Supplementary Material

Additional File 1 normative model of a primary health care system developed for this study Click here for file
