# Supplementary material for: Developments in Australian general practice 2000–2002: what did these contribute to a well functioning and comprehensive Primary Health Care System?
Source: Aust New Zealand Health Policy. 2006 Jan 15;3:1. doi: 10.1186/1743-8462-3-1 (PMC1379649; doi:10.1186/1743-8462-3-1)
Supplement: Additional File 1 — normative model of a primary health care system developed for this study [file 1743-8462-3-1-S1.doc]

## Conceptual framework for a primary health care system

Figure 4 shows the normative model of a primary health care system developed for this study, based on a literature review and consultations with an expert panel convened for the project.

**Figure 4: Primary Health Care Framework**

| **CONTEXTUAL ISSUES** | | | | | |
| --- | --- | --- | --- | --- | --- |
| **GOALS & VALUES** | **SERVICE PLANNING & DEVELOPMENT** | **CORE FUNCTIONS to INDIVIDUALS & POPULATIONS** | **APPROACHES** | **INDICATORS** | **OUTCOMES** |
|  | **CAPACITY** | | |  |  |
| **LEVELS OF OPERATION**  **CONSUMERS**  (e.g. individual consumers, carers, consumer groups)  **PRACTITIONERS**  (e.g. GPs, community health nurses, allied health professionals)  **PRACTICES/COMMUNITY HEALTH TEAMS**  (within a Community Health Service area)  **SUB-REGIONAL**  (e.g. Division of General Practice, community health services, NGOs)  **REGIONAL**  (e.g. Area/District Health Services)  **STATE**  (e.g. SBOs, State/Territory Department, State/Territory Offices of DoHA)  **NATIONAL**  (e.g Departments of Health and Ageing, Veterans’ Affairs | | | | | |

Within this model, the *contextual issues* are the features of the Australian health care system that effect how primary health care operates and its opportunities for development. The *goals and values* include the broad goals to which primary health care organisations, services and initiatives contribute and the values that underlie these goals. *Service planning and development* includes the tasks which are required to develop and maintain effective services, including setting priorities, clarifying roles and responsibilities within and between services, and developing service plans.

The *core functions* are the types of primary health care services needed to meet the health needs of the population. These include both services to individuals and population based services. Functions directed to individuals include:

- promoting normal development and functioning;
- prevention, early detection and management of health risk factors;
- providing episodic care for undifferentiated presentations;
- assessing, triaging and referring appropriately within health and other human services;
- facilitating and/or coordinating care across multiple providers;
- providing ongoing care of chronic and complex health problems, including physical, psychosocial, post-acute and rehabilitative aspects throughout the lifecycle;
- advocating for individuals and groups of individuals with similar concerns.

Functions directed to populations include:

- supporting population focused programs to prevent and control communicable and non-communicable diseases;
- strengthening community capacity to identify and address problems, through community development, participation & empowerment;
- contributing to actions which ensure safe and healthy environments, including working in settings such as schools and workplaces, and supporting initiatives by other sectors;
- advocating for healthy public policy, through raising awareness of issues, and involvement as a key stakeholder in policy development.

*Approaches* refers to ways of providing or organising health care which are characteristic of primary health care and are often believed to contribute to its effectiveness. They include a socio-ecologic view of health, multidisciplinary teamwork, intersectoral collaboration, consumer and community participation, and the use of multiple and systematic strategies.

*Capacity* covers the elements required to maintain an effective, adaptable and sustainable PHC system. These include:

- workforce supply, education, training and development, and distribution;
- leadership in PHC;
- funding arrangements and remuneration which provide appropriate incentives;
- coherent and supportive policy;
- organisational structures;
- partnerships and inter-organisational networks;
- facilities & equipment; and
- IM/IT systems.

The *outcomes* that are relevant to primary health care systems relate to health status, equity, patient and provider satisfaction, costs and efficiency. *Indicators* are used to measure achievement of primary health care outcomes, and may relate to qualities such as accessibility, appropriateness, comprehensiveness, effectiveness, efficiency, equity, safety, and sustainability. Some of these are reflected in the National Health Performance Framework [9].

The model was generally well received by participants in the study, with the majority finding it comprehensive, representative of and relevant to their work. The most common criticism was that consumers were not explicitly recognised in the original version. The model was used to map the focus of initiatives and identify areas which have been relatively under-developed.
